# κ-opioid receptor is involved in the cardioprotection induced by exercise training

**DOI:** 10.1371/journal.pone.0170463

**Published:** 2017-03-16

**Authors:** Xiao Geng, Honglin Zhao, Shumiao Zhang, Juan Li, Fei Tian, Na Feng, Rong Fan, Min Jia, Haitao Guo, Liang Cheng, Jincheng Liu, Wensheng Chen, Jianming Pei

**Affiliations:** 1 Department of Physiology, National Key Discipline of Cell Biology, Fourth Military Medical University, Xi’an, Shaanxi Province, People’s Republic of China; 2 Department of Physical Education, Chang’an University, Xi'an, Shaanxi Province, China; 3 Department of Cardiovascular Surgery, First Hospital of Lanzhou University, Lanzhou, Gansu Province, China; 4 Department of Cardiovascular Surgery, Xijing Hospital, Fourth Military Medical University, Xi’an, Shaanxi Province, China; Indiana University School of Medicine, UNITED STATES

## Abstract

The present study was designed to test the hypothesis that exercise training elicited a cardioprotective effect against ischemia and reperfusion (I/R) via the κ-opioid receptor (κ-OR)-mediated signaling pathway. Rats were randomly divided into four groups: the control group, the moderate intensity exercise (ME) group, the high intensity exercise (HE) group, and the acute exercise (AE) group. For the exercise training protocols, the rats were subjected to one week of adaptive treadmill training, while from the second week, the ME and HE groups were subjected to eight weeks of exercise training, and the AE group was subjected to three days of adaptive treadmill training and one day of vigorous exercise. After these protocols, the three exercise training groups were divided into different treatment groups, and the rats were subjected to 30 min of ischemia and 120 min of reperfusion. Changes in infarct size and serum cTnT (cardiac troponin T) caused by I/R were reduced by exercise training. Moreover, cardiac dysfunction caused by I/R was also alleviated by exercise training. These effects of exercise training were reversed by nor-BNI (a selective κ-OR antagonist), Compound C (a selective AMPK inhibitor), Akt inhibitor and L-NAME (a non-selective eNOS inhibitor). Expression of κ-OR and phosphorylation of AMPK, Akt and eNOS were significantly increased in the ME, HE and AE groups. These findings demonstrated that the cardioprotective effect of exercise training is possibly mediated by the κ-OR-AMPK-Akt-eNOS signaling pathway.

## Introduction

In 1889, Loomis found a relationship between physical exercise, as a type of treatment, and heart diseases [1]. A large number of studies have demonstrated that exercise training is associated with improvement of the cardiac function of ischemia-reperfusion (I/R)-affected myocardium [2,3,4,5]. Myocardium depends on expansion of the coronary artery for more blood and oxygen supplementation when subjected to ischemia or hypoxia. After long-term exercise training, myocardial collateral circulation is established, and capillaries are proliferated [6]. This effect is one of the reasons for the prevention of myocardial infarction caused by ischemia [7]. Exercise training improves oxidation resistance and resistance of apoptosis, leading to production of some proteins and mediators of cardioprotection and contributing to cardioprotective effects [5,8,9,10,11,12,13]. It has been demonstrated that different modes and intensities of exercise training can mediate cardioprotection in ischemic myocardium [14,15,16,17,18,19]. However, the mechanism underlying cardioprotection from exercise training still warrants further study.

As with exercise training, many types of cardioprotective effects have been identified. For example, it has been demonstrated that U50,488H, a selective agonist of κ-OR, reduces the myocardial infarct size caused by I/R [20,21,22,23], and this effect is reversed by nor-BNI, which is a selective antagonist of κ-OR [22,24,25]. Moreover, several studies have shown that U50,488H increases the expression levels and phosphorylation of Akt and eNOS and NO production, as well as reducing myocardial apoptosis [26,27]. However, little is known about the relationship of cardioprotective mechanisms from exercise training and the κ-OR system.

It has been reported that, after short-term high intensity treadmill training, the mRNAs of opioid receptors are increased, and the myocardial infarct size is reduced [16]. In addition, the cardioprotection conferred by exercise training is blunted by blockade of the opioid system [28]. Recently, Miller et al. and Borges et al. demonstrated that delta-OR partly exerted cardioprotection against necrosis [29,30]. It seems that the opioid system might be involved in the cardioprotection induced by exercise training. Further results have also revealed that morphine-induced cardioprotection occurs via an AMPK-dependent signaling pathway [31,32]. It is known that AMPK is an important signaling molecule in cardioprotection. The stimulation of AMPK and Akt has a very close relationship with the effect of attenuating I/R injury by ischemic preconditioning [31]. In I/R hearts, the activation of AMPK is an adaptive response of the heart; it provides ATP to protect myocardial tissue from damage during hypoxia [32,33,34]. Exercise training stimulates myocardial AMPK to regulate cellular metabolism [35,36], and different exercise intensities and durations also elicit the stimulation of AMPK.

Together, these data suggested that both exercise training and the OR system could provide cardioprotective effects. There are at least three types of isotypes of OR in cardiovascular system, and κ-OR is a predominant subtype in the heart [37]. In the present study, we tested the hypothesis that theκ-OR-mediated AMPK-Akt-eNOS signaling pathway was involved in cardioprotection induced by exercise training. In the presence and absence of different blockers, we used three exercise training protocols and an I/R animal model to determine the changes in hemodynamics, the expression of κ-OR, and the phosphorylation of AMPK, Akt and eNOS. We also examined myocardial infarct size and serum cTnT and NO production. Data from the present study indicated that the cardioprotective effect of exercise training was mediated by the κ-OR-AMPK-Akt-eNOS signaling pathway.

## Materials and methods

### Animals

The rats (n = 168) were purchased from the animal center of the Fourth Military Medical University. All of the animals used in this study were cared for in accordance with the Guide for the Care and Use of Laboratory Animals published by the United States National Institutes of Health (NIH publication no. 85–23, revised 1996), and all of the procedures were approved by the Committee of Experimental Animals of the Fourth Military Medical University.

### Experimental protocol

1. Male Sprague-Dawley rats (200-250g) were randomized to a sham exercise training control group (placed on a treadmill without belt movement) or three exercise training groups (ME group, HE group, AE group; n = 8). For the ME and HE groups, in the first week, all of the rats were exercised in the morning for 30 min on the treadmill (Shanghai Jide equipment factory) at the speed of 15m/min for five consecutive days. From the second week, the rats were exercised at the speed of 20m/min, with the exercise period lasting 30 min. The exercise period daily increased by 10min from 30min to 60min. The training speed of the ME group was constant, and that of the HE group increased to 30m/min from the third week. All of the rats were exercised for eight weeks. For the AE group, the first three days for this group as described above were used to train and prepare the animals for a vigorous period of exercise occurring the morning of day four, which consisted of an exercise period lasting for 60 min at 30 m/min[38].
2. The three exercise training groups were divided into different treatment groups (n = 8), including the sham group; I/R—left coronary artery was ligated for 30 min and then was allowed to undergo 120 min of reperfusion(I/R, see details below) with receiving vehicle (0.9% NaCl, iv.) and the I/R+(ME, HE, AE) groups: the I/R+(ME, HE, AE)+nor-BNI group—nor-BNI, a selectiveκ-OR antagonist (Tocris, USA, 2 mg/kg, iv.) was administered 20 min before reperfusion; the I/R+(ME, HE, AE)+Compound C group—Compound C, an AMPK inhibitor (Abcam, United Kingdom, 20mg/kg, iv.) was administered 20 min before reperfusion; the I/R+(ME, HE, AE)+Akt inhibitor group—Akt inhibitor (Calbiochem, China,0.3mg/kg, i.v.) was administered 20 min before reperfusion; and the I/R+(ME, HE, AE)+L-NAME group—L-NAME, an eNOS inhibitor (Calbiochem, China, 30mg/kg, iv.), was injected 20 min before reperfusion.

### Model of ischemia-reperfusion (I/R) injury

At the end of the exercise training protocols, the rats were administered 3% pentobarbital (2 ml/kg, i.p.). An incision was made on the left thorax, and then a slip (5–0 silk) was threaded around the left coronary artery, approximately 2 mm from the edge of the auricula sinistra. Myocardial ischemia was produced by making a slipknot for 30 min. After ischemia, the animal experienced 120 min of reperfusion.

### Determination of cTnT Efflux

Blood samples were collected at the end of reperfusion and were centrifuged to obtain plasma. Then, cTnT was determined by ELISA. All of the steps followed the kit instructions strictly.

### Determination of myocardial infarct size

**Fig 1:**
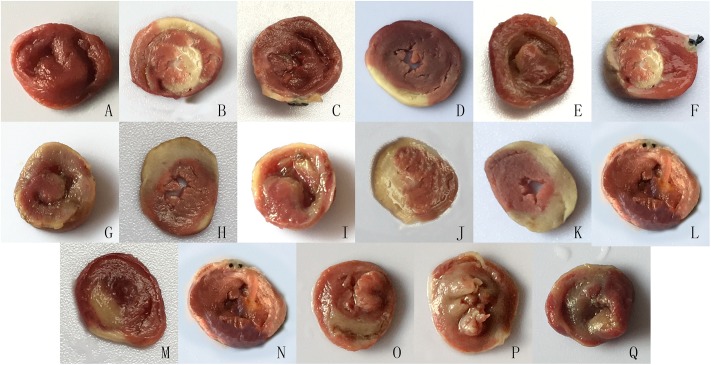
Representative photographs of triphenyltetrazolium chloride (TTC)-stained left ventricular tissue. (A) Sham; (B) I/R, 30 min of ischemia and 120 min of reperfusion; (C) I/R + ME, moderate intensity exercise for 8 wk before I/R; (D) I/R + HE, high intensity exercise for 8 wk before I/R; (E) I/R + AE, acute exercise before I/R; (F) I/R + ME+ nor-BNI; (G) I/R + HE + nor-BNI; (H) I/R + AE + nor-BNI; (I) I/R + ME + Compound C; (J) I/R + HE + Compound C; (K) I/R + AE + Compound C; (L) I/R + ME + Akt inhibitor; (M) I/R + HE + Akt inhibitor; (N) I/R + AE + Akt inhibitor; (O) I/R + ME + L-NAME; (P) I/R + HE + L-NAME; (Q) I/R + AE + L-NAME. In each study, nor-BNI (a selective κ-OR antagonist, 2.0 mg/kg), Compound C (an AMPK inhibitor, 20 mg/kg), Akt inhibitor (0.3 mg/kg) and L-NAME (an eNOS inhibitor, 30 mg/kg) were administered 20min before reperfusion. Left ventricular tissue was then processed and stained with TTC to determine the viable (red) and nonviable (white) myocardium. Infarct sizes were determined as described in the Methods section.

At the end of reperfusion, the heart was harvested, washed in saline immediately, and frozen at -20°C for 2 h subsequently. The heart was sliced transversely from apex to base into2-mm cross-sections and was placed in 2% TTC phosphate buffer for 15 min at 37°C. Myocardial infarct size was determined by means of a TTC staining technique (infarct area/area at risk×100%) as described previously. The ischemic area was dyed red by TTC staining; and the myocardial infarction-affected part could not be TTC stained ad thus appeared white. The hearts were photographed (Fig 1) finally.

### Western blot analysis for expression of κ-OR and AMPK, Akt, and eNOS phosphorylation

Proteins were extracted from heart tissue and myocytes. Total protein concentrations of the samples were tested using a BCA protein assay kit. The immunoblots were probed with the following antibodies overnight at 4°C: anti-κ-OR (Santa Cruz, USA), anti-pAMPK, anti-pAkt, and anti-peNOS (Cell Signaling, Beverly, USA). The corresponding secondary antibodies were incubated at room temperature for 1 h subsequently. Band intensities were quantified by densitometry.

### Statistical analysis

All of the data are expressed as the mean ± SEM. Hemodynamics data were analyzed with the chi-square test and ANOVA. After analysis by ANOVA, Bonferroni’s correction was conducted for post hoc *t*-tests. Infarct size, cTnT and NO from each group were compared by ANOVA. *P*<0.05 was considered to be statistically significant.

## Results

### Cardioprotection of exercise training in rats

**Fig 2:**
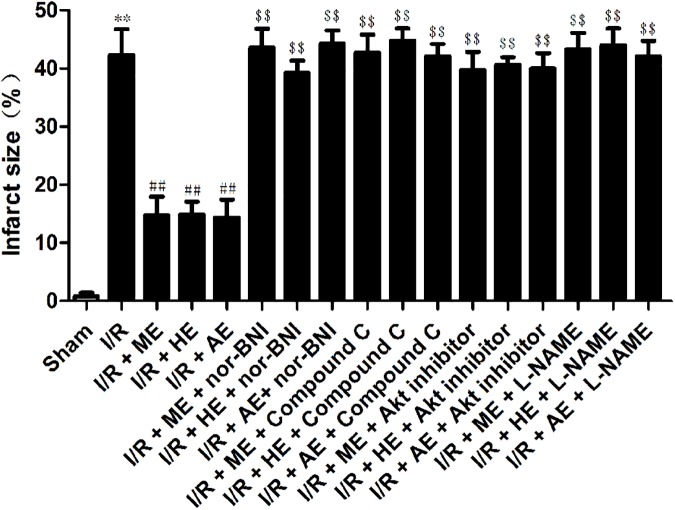
Effects of exercise training on infarct size after I/R. **Values are expressed as %infarct size.** All of the results are expressed as the mean ± SEM. I/R, 30 min of ischemia and 120 min of reperfusion; I/R + ME, moderate intensity exercise for 8 wk before I/R; I/R + HE, high intensity exercise for 8 wk before I/R; I/R + AE, acute exercise before I/R; I/R + ME+ nor-BNI, moderate intensity exercise for 8 wk before I/R plus nor-BNI (2.0 mg/kg); I/R + HE + nor-BNI, high intensity exercise for 8 wk before I/R plus nor-BNI (2.0 mg/kg); I/R + AE + nor-BNI; acute exercise before I/R plus nor-BNI (2.0 mg/kg); I/R + ME + Compound C, moderate intensity exercise for 8 wk before I/R plus Compound C (20 mg/kg); I/R + HE + Compound C, high intensity exercise for 8 wk before I/R plus Compound C (20 mg/kg); I/R + AE + Compound C, acute exercise before I/R plus Compound C (20 mg/kg); I/R + ME + Akt inhibitor, moderate intensity exercise for 8 wk before I/R plus Akt inhibitor (0.3 mg/kg); I/R + HE + Akt inhibitor, high intensity exercise for 8 wk before I/R plus Akt inhibitor (0.3 mg/kg); I/R + AE + Akt inhibitor, acute exercise before I/R plus Akt inhibitor (0.3 mg/kg); I/R + ME + L-NAME, moderate intensity exercise for 8 wk before I/R plus L-NAME (30 mg/kg); I/R + HE + L-NAME, high intensity exercise for 8 wk before I/R plus L-NAME (30 mg/kg); I/R + AE + L-NAME, acute exercise before I/R plus L-NAME (30 mg/kg). n = 8, **P<0.01 vs. sham, ^##^*P*<0.01 vs. I/R, ^$$^ *P*<0.01 vs. I/R+ME, I/R+HE, I/R+AE.

**Fig 3:**
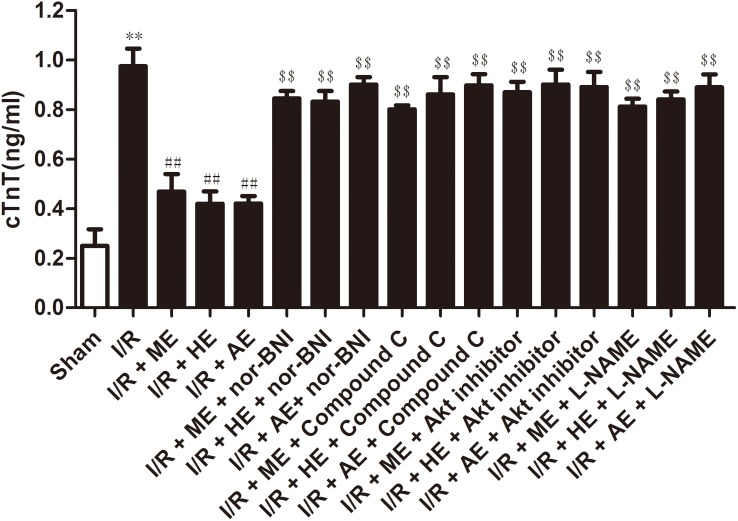
Effects of exercise training on cTnT in serum after I/R. All results are expressed as the means ± SEM.I/R, 30 min ischemia and 120 min reperfusion; I/R + ME, moderate intensity exercise for 8 wk before I/R; I/R + HE, high intensity exercise for 8 wk before I/R; I/R + AE, acute exercise before I/R; I/R + ME+ nor-BNI, moderate intensity exercise for 8 wk before I/R plus nor-BNI (2.0 mg/kg); I/R + HE + nor-BNI, high intensity exercise for 8 wk before I/R plus nor-BNI (2.0 mg/kg); I/R + AE + nor-BNI; acute exercise before I/R plus nor-BNI (2.0 mg/kg); I/R + ME + Compound C, moderate intensity exercise for 8 wk before I/R plus Compound C (20 mg/kg); I/R + HE + Compound C, high intensity exercise for 8 wk before I/R plus Compound C (20 mg/kg); I/R + AE + Compound C, acute exercise before I/R plus Compound C (20 mg/kg); I/R + ME + Akt inhibitor, moderate intensity exercise for 8 wk before I/R plus Akt inhibitor (0.3 mg/kg); I/R + HE + Akt inhibitor, high intensity exercise for 8 wk before I/R plus Akt inhibitor (0.3 mg/kg); I/R + AE + Akt inhibitor, acute exercise before I/R plus Akt inhibitor (0.3 mg/kg); I/R + ME + L-NAME, moderate intensity exercise for 8 wk before I/R plus L-NAME (30 mg/kg); I/R + HE + L-NAME, high intensity exercise for 8 wk before I/R plus L-NAME (30 mg/kg); I/R + AE + L-NAME, acute exercise before I/R plus L-NAME (30 mg/kg). n = 8, **P<0.01 vs. sham, ^##^*P*<0.01 vs. I/R, ^$$^*P*<0.01 vs. I/R+ME, I/R+HE, I/R+AE, correspondingly.

After the exercise training protocols, the rats were treated with 30 min of ischemia and 120 min of reperfusion. I/R induced injury to myocardial tissue; 30 min of ischemia followed by 120 min of reperfusion resulted in a mean infarct size of 42.3±4% (*P*<0.01; Fig 2). The exercise training groups experienced significant cardioprotective effects in I/R myocardial tissue (infarct size was 24.7±3% in the I/R+ME group; 24.9±2% in the I/R+HE group; 24.4±3% in the I/R+AE group; *P*<0.01; Fig 2). In addition, in the I/R group, serum cTnT increased sharply after I/R (*P*<0.01; Fig 3), and this phenomenon was significantly attenuated by exercise training (*P*<0.01; Fig 3). The above cardioprotective effects of exercise training were blocked by nor-BNI, Compound C, Akt inhibitor and L-NAME.

### Effects of exercise training on cardiac hemodynamics

We observed a significant reduction in HR in the I/R group and all of the exercise training groups by the chi-square test; however, at the same time point, we found no significant differences among the three exercise training groups (Table 1). In the I/R group, compared with the sham group, cardiac function significantly decreased. The LVP in each exercise training group underwent a significant increase, compared with the I/R group (Table 2). Similarly, significant differences were found in ±dP/dtmax between the exercise training hearts and I/R hearts (Tables 3 and 4). However, these effects of exercise training on hemodynamics were reversed by nor-BNI, Compound C, Akt inhibitor and L-NAME.

**Table 1:** Changes in heart rate (bpm) during myocardial I/R.

	Baseline	Ischemia30min	Reperfusion(min)	
			60	120
**Sham**	404±2	402±4	394±3	388±3
**I/R**	398±3	362±4**^●●^	355±3**^●●^	356±2**^●●^
**I/R + ME**	402±6	366±4^●●^	362±2^●●^	353±4^●●^
**I/R + HE**	409±4	364±5^●●^	359±5^●●^	350±3^●●^
**I/R + AE**	411±3	369±5^●●^	355±5^●●^	354±3^●●^
**I/R + ME + nor-BNI**	404±4	368±4^●●^	365±3^●●^	360±4^●●^
**I/R + HE + nor-BNI**	405±7	371±3^●●^	365±4^●●^	360±4^●●^
**I/R + AE + nor-BNI**	403±4	370±7^●●^	358±3^●●^	350±3^●●^
**I/R + ME + Compound C**	407±4	374±5^●●^	372±3^●●^	362±3^●●^
**I/R + HE + Compound C**	402±5	374±5^●●^	362±3^●●^	356±3^●●^
**I/R + AE + Compound C**	401±3	377±6^●●^	363±4^●●^	354±3^●●^
**I/R + ME + Akt inhibitor**	407±4	374±3^●●^	364±5^●●^	351±4^●●^
**I/R + HE + Akt inhibitor**	406±4	369±3^●●^	363±2^●●^	358±1^●●^
**I/R + AE + Akt inhibitor**	404±2	367±3^●●^	361±1^●●^	354±3^●●^
**I/R + ME + L-NAME**	399±4	366±2^●●^	353±3^●●^	353±4^●●^
**I/R + HE + L-NAME**	403±3	373±2^●●^	364±2^●●^	351±4^●●^
**I/R + AE + L-NAME**	403±2	371±2^●●^	367±2^●●^	359±4^●●^

All of the results are expressed as the means ± SEMs. I/R, 30 min of ischemia and 120 min of reperfusion; I/R + ME, moderate intensity exercise for 8 wk before I/R; I/R + HE, high intensity exercise for 8 wk before I/R; I/R + AE, acute exercise before I/R; I/R + ME+ nor-BNI, moderate intensity exercise for 8 wk before I/R plus nor-BNI (2.0 mg/kg); I/R + HE + nor-BNI, high intensity exercise for 8 wk before I/R plus nor-BNI (2.0 mg/kg); I/R + AE + nor-BNI; acute exercise before I/R plus nor-BNI (2.0 mg/kg); I/R + ME + Compound C, moderate intensity exercise for 8 wk before I/R plus Compound C (20 mg/kg); I/R + HE + Compound C, high intensity exercise for 8 wk before I/R plus Compound C (20 mg/kg); I/R + AE + Compound C, acute exercise before I/R plus Compound C (20 mg/kg); I/R + ME + Akt inhibitor, moderate intensity exercise for 8 wk before I/R plus Akt inhibitor (0.3 mg/kg); I/R + HE + Akt inhibitor, high intensity exercise for 8 wk before I/R plus Akt inhibitor (0.3 mg/kg); I/R + AE + Akt inhibitor, acute exercise before I/R plus Akt inhibitor (0.3 mg/kg); I/R + ME + L-NAME, moderate intensity exercise for 8 wk before I/R plus L-NAME (30 mg/kg); I/R + HE + L-NAME, high intensity exercise for 8 wk before I/R plus L-NAME (30 mg/kg); I/R + AE + L-NAME, acute exercise before I/R plus L-NAME (30 mg/kg). n = 8

**P<0.01 vs. sham

^●●^*P*<0.01 vs. baseline.

**Table 2:** Changes in LVP (mmHg) during myocardial I/R.

	Baseline	Ischemia30min	Reperfusion(min)	
			60	120
**Sham**	127±3	121±2	123±2	120±2
**I/R**	134±1	95±2**^●●^	83±1**^●●^	70±2**^●●^
**I/R + ME**	130±1	115±2^##^^●●^	110±2^##^^●●^	102±2^##^^●●^
**I/R + HE**	131±2	109±3^##^^●●^	102±2^##^^●●^	99±3^##^^●●^
**I/R + AE**	133±2	112±3^##^^●●^	107±4^##^^●●^	102±2^##^^●●^
**I/R + ME + nor-BNI**	127±2	90±2^$$^^●●^	85±3^$$^^●●^	67±2^$$^^●●^
**I/R + HE + nor-BNI**	127±2	94±3^$$^^●●^	82±3^$$^^●●^	69±3^$$^^●●^
**I/R + AE + nor-BNI**	132±2	90±3^$$^^●●^	83±2^$$^^●●^	72±1^$$^^●●^
**I/R + ME + Compound C**	130±2	92±2^$$^^●●^	80±3^$$^^●●^	69±1^$$^^●●^
**I/R + HE + Compound C**	130±4	92±3^$$^^●●^	76±3^$$^^●●^	73±4^$$^^●●^
**I/R + AE + Compound C**	128±2	90±4^$$^^●●^	83±2^$$^^●●^	69±3^$$^^●●^
**I/R + ME + Akt inhibitor**	129±2	92±3^$$^^●●^	82±2^$$^^●●^	71±2^$$^^●●^
**I/R + HE + Akt inhibitor**	128±2	87±2^$$^^●●^	75±2^$$^^●●^	72±2^$$^^●●^
**I/R + AE + Akt inhibitor**	130±2	88±2^$$^^●●^	82±1^$$^^●●^	69±2^$$^^●●^
**I/R + ME + L-NAME**	131±1	88±2^$$^^●●^	80±2^$$^^●●^	66±3^$$^^●●^
**I/R + HE + L-NAME**	132±2	89±3^$$^^●●^	78±2^$$^^●●^	72±2^$$^^●●^
**I/R + AE + L-NAME**	129±2	91±3^$$^^●●^	81±1^$$^^●●^	70±2^$$^^●●^

All of the results are expressed as the means ± SEMs. I/R, 30 min of ischemia and 120 min of reperfusion; I/R + ME, moderate intensity exercise for 8 wk before I/R; I/R + HE, high intensity exercise for 8 wk before I/R; I/R + AE, acute exercise before I/R; I/R + ME+ nor-BNI, moderate intensity exercise for 8 wk before I/R plus nor-BNI (2.0 mg/kg); I/R + HE + nor-BNI, high intensity exercise for 8 wk before I/R plus nor-BNI (2.0 mg/kg); I/R + AE + nor-BNI; acute exercise before I/R plus nor-BNI (2.0 mg/kg); I/R + ME + Compound C, moderate intensity exercise for 8 wk before I/R plus Compound C (20 mg/kg); I/R + HE + Compound C, high intensity exercise for 8 wk before I/R plus Compound C (20 mg/kg); I/R + AE + Compound C, acute exercise before I/R plus Compound C (20 mg/kg); I/R + ME + Akt inhibitor, moderate intensity exercise for 8 wk before I/R plus Akt inhibitor (0.3 mg/kg); I/R + HE + Akt inhibitor, high intensity exercise for 8 wk before I/R plus Akt inhibitor (0.3 mg/kg); I/R + AE + Akt inhibitor, acute exercise before I/R plus Akt inhibitor (0.3 mg/kg); I/R + ME + L-NAME, moderate intensity exercise for 8 wk before I/R plus L-NAME (30 mg/kg); I/R + HE + L-NAME, high intensity exercise for 8 wk before I/R plus L-NAME (30 mg/kg); I/R + AE + L-NAME, acute exercise before I/R plus L-NAME (30 mg/kg). n = 8

**P<0.01 vs. sham

^##^*P*<0.01 vs. I/R

^$$^*P*<0.01 vs. I/R+ME, I/R+HE, and I/R+AE

^●●^*P*<0.01 vs. baseline.

**Table 3:** Changes in dP/dtmax (mm Hg/s) during myocardial I/R.

	Baseline	Ischemia 30min	Reperfusion(min)	
			60	120
**Sham**	3980±47	3917±23	3908±21	3902±23
**I/R**	4102±33	2225±67**^●●^	1881±51**^●●^	1621±56**^●●^
**I/R + ME**	3978±37	2822±47^##^^●●^	2626±48^##^^●●^	2487±56^##^^●●^
**I/R + HE**	3902±32	3013±98^##^^●●^	2798±53^##^^●●^	2544±58^##^^●●^
**I/R + AE**	3999±69	2994±57^##^^●●^	2605±54^##^^●●^	2575±57^##^^●●^
**I/R + ME + nor-BNI**	3985±34	2174±58^$$^^●●^	1929±46^$$^^●●^	1701±40^$$^^●●^
**I/R + HE + nor-BNI**	3945±29	2054±30^$$^^●●^	1939±38^$$^^●●^	1681±62^$$^^●●^
**I/R + AE + nor-BNI**	4013±47	2201±33^$$^^●●^	1892±36^$$^^●●^	1667±47^$$^^●●^
**I/R + ME + Compound C**	3967±45	2068±79^$$^^●●^	1852±52^$$^^●●^	1622±38^$$^^●●^
**I/R + HE + Compound C**	3967±41	2004±29^$$^^●●^	1952±45^$$^^●●^	1722±46^$$^^●●^
**I/R + AE + Compound C**	4088±55	2145±44^$$^^●●^	1897±34^$$^^●●^	1709±36^$$^^●●^
**I/R + ME + Akt inhibitor**	3985±27	2299±56^$$^^●●^	1993±62^$$^^●●^	1712±43^$$^^●●^
**I/R + HE + Akt inhibitor**	3916±29	2089±66^$$^^●●^	1803±35^$$^^●●^	1667±27^$$^^●●^
**I/R + AE + Akt inhibitor**	4057±46	2112±34^$$^^●●^	1904±37^$$^^●●^	1709±38^$$^^●●^
**I/R + ME + L-NAME**	3956±31	2223±56^$$^^●●^	1915±36^$$^^●●^	1696±34^$$^^●●^
**I/R + HE + L-NAME**	3923±45	2105±35^$$^^●●^	1808±27^$$^^●●^	1614±19^$$^^●●^
**I/R + AE + L-NAME**	3998±44	2199±38^$$^^●●^	1986±39^$$^^●●^	1711±35^$$^^●●^

All of the results are expressed as the means ± SEMs. I/R, 30 min of ischemia and 120 min of reperfusion; I/R + ME, moderate intensity exercise for 8 wk before I/R; I/R + HE, high intensity exercise for 8 wk before I/R; I/R + AE, acute exercise before I/R; I/R + ME+ nor-BNI, moderate intensity exercise for 8 wk before I/R plus nor-BNI (2.0 mg/kg); I/R + HE + nor-BNI, high intensity exercise for 8 wk before I/R plus nor-BNI (2.0 mg/kg); I/R + AE + nor-BNI; acute exercise before I/R plus nor-BNI (2.0 mg/kg); I/R + ME + Compound C, moderate intensity exercise for 8 wk before I/R plus Compound C (20 mg/kg); I/R + HE + Compound C, high intensity exercise for 8 wk before I/R plus Compound C (20 mg/kg); I/R + AE + Compound C, acute exercise before I/R plus Compound C (20 mg/kg); I/R + ME + Akt inhibitor, moderate intensity exercise for 8 wk before I/R plus Akt inhibitor (0.3 mg/kg); I/R + HE + Akt inhibitor, high intensity exercise for 8 wk before I/R plus Akt inhibitor (0.3 mg/kg); I/R + AE + Akt inhibitor, acute exercise before I/R plus Akt inhibitor (0.3 mg/kg); I/R + ME + L-NAME, moderate intensity exercise for 8 wk before I/R plus L-NAME (30 mg/kg); I/R + HE + L-NAME, high intensity exercise for 8 wk before I/R plus L-NAME (30 mg/kg); I/R + AE + L-NAME, acute exercise before I/R plus L-NAME (30 mg/kg). n = 8

**P<0.01 vs. sham

^##^*P*<0.01 vs. I/R

^$$^*P*<0.01 vs. I/R+ME, I/R+HE, and I/R+AE.

^●●^*P*<0.01 vs. baseline.

**Table 4:** Changes in -dP/dtmax (mm Hg/s) during myocardial I/R.

	Baseline	Ischemia 30min	Reperfusion(min)	
			60	120
**Sham**	-2780±12	-2680±16	-2662±18	-2623±24
**I/R**	-2664±35	-1751±17**^●●^	-1546±35**^●●^	-1224±37**^●●^
**I/R + ME**	-2774±20	-2349±28^##^^●●^	-1921±26^##^^●●^	-1737±29^##^^●●^
**I/R + HE**	-2701±36	-2268±63^##^^●●^	-1923±56^##^^●●^	-1803±35^##^^●●^
**I/R + AE**	-2768±23	-2299±55^##^^●●^	-1901±35^##^^●●^	-1721±46^##^^●●^
**I/R + ME+ nor-BNI**	-2689±18	-1845±44^$$^^●●^	-1530±29^$$^^●●^	-1213±36^$$^^●●^
**I/R + HE + nor-BNI**	-2699±26	-1877±84^$$^^●●^	-1630±48^$$^^●●^	-1313±46^$$^^●●^
**I/R + AE + nor-BNI**	-2733±35	-1744±69^$$^^●●^	-1564±54^$$^^●●^	-1232±46^$$^^●●^
**I/R + ME + Compound C**	-2699±41	-1775±55^$$^^●●^	-1541±64^$$^^●●^	-1116±45^$$^^●●^
**I/R + HE + Compound C**	-2699±34	-1765±49^$$^^●●^	-1641±37^$$^^●●^	-1216±40^$$^^●●^
**I/R + AE + Compound C**	-2680±54	-1789±51^$$^^●●^	-1522±50^$$^^●●^	-1276±45^$$^^●●^
**I/R + ME + Akt inhibitor**	-2683±40	-1876±43^$$^^●●^	-1643±54^$$^^●●^	-1223±35^$$^^●●^
**I/R + HE + Akt inhibitor**	-2713±42	-1834±46^$$^^●●^	-1622±56^$$^^●●^	-1211±47^$$^^●●^
**I/R + AE + Akt inhibitor**	-2733±47	-1789±39^$$^^●●^	-1567±56^$$^^●●^	-1189±49^$$^^●●^
**I/R + ME + L-NAME**	-2731±38	-1801±45^$$^^●●^	-1622±27^$$^^●●^	-1308±14^$$^^●●^
**I/R + HE + L-NAME**	-2723±62	-1719±46^$$^^●●^	-1514±49^$$^^●●^	-1285±54^$$^^●●^
**I/R + AE + L-NAME**	-2701±60	-1689±42^$$^^●●^	-1541±47^$$^^●●^	-1256±38^$$^^●●^

All of the results are expressed as the means ± SEMs. I/R, 30 min of ischemia and 120 min of reperfusion; I/R + ME, moderate intensity exercise for 8 wk before I/R; I/R + HE, high intensity exercise for 8 wk before I/R; I/R + AE, acute exercise before I/R; I/R + ME+ nor-BNI, moderate intensity exercise for 8 wk before I/R plus nor-BNI (2.0 mg/kg); I/R + HE + nor-BNI, high intensity exercise for 8 wk before I/R plus nor-BNI (2.0 mg/kg); I/R + AE + nor-BNI; acute exercise before I/R plus nor-BNI (2.0 mg/kg); I/R + ME + Compound C, moderate intensity exercise for 8 wk before I/R plus Compound C (20 mg/kg); I/R + HE + Compound C, high intensity exercise for 8 wk before I/R plus Compound C (20 mg/kg); I/R + AE + Compound C, acute exercise before I/R plus Compound C (20 mg/kg); I/R + ME + Akt inhibitor, moderate intensity exercise for 8 wk before I/R plus Akt inhibitor (0.3 mg/kg); I/R + HE + Akt inhibitor, high intensity exercise for 8 wk before I/R plus Akt inhibitor (0.3 mg/kg); I/R + AE + Akt inhibitor, acute exercise before I/R plus Akt inhibitor (0.3 mg/kg); I/R + ME + L-NAME, moderate intensity exercise for 8 wk before I/R plus L-NAME (30 mg/kg); I/R + HE + L-NAME, high intensity exercise for 8 wk before I/R plus L-NAME (30 mg/kg); I/R + AE + L-NAME, acute exercise before I/R plus L-NAME (30 mg/kg). n = 8

**P<0.01 vs. sham

^##^*P*<0.01 vs. I/R

^$$^*P*<0.01 vs. I/R+ME, I/R+HE, and I/R+AE.

^●●^*P*<0.01 vs. baseline.

### Relationship between exercise training and signaling molecules in rats

**Fig 4:**
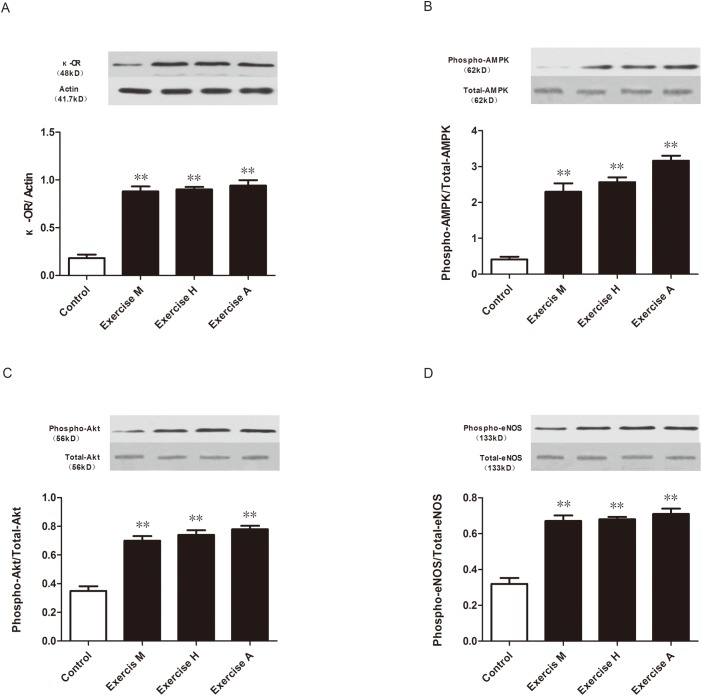
**Effects of exercise training with different intensities on expression of** κ**-OR (A), AMPK (B), Akt (C) and eNOS (D)**. All of the results are expressed as the means ± SEMs. ME, moderate intensity exercise for 8 wk; HE, high intensity exercise for 8 wk; AE, acute exercise. n = 8, **P<0.01 vs. control.

We designed the initial studies to confirm that exercise training resulted in the changes in expression levels of signaling molecules. In this set of experiments, two groups (n = 8) of rats underwent the long-term exercise training protocol, eight rats underwent the acute exercise training protocol, and eight rats served as the controls. The results from these experiments revealed that exercise training associated with significant up-regulation of the expression of κ-OR and phosphorylation of AMPK, Akt and eNOS (Fig 4). Using the three exercise training protocols, we found no significant differences in expression levels of the above molecules among these exercise protocols.

## Discussion

There were several observations in this study. First, the cardioprotective effects of exercise training were manifested as ameliorating the dysfunction of hemodynamics and reducing the myocardial infarct size and serum cTnT after myocardial I/R. The cardioprotection gained by exercise training was reversed by nor-BNI, Compound C, Akt inhibitor and L-NAME. Second, expression of κ-OR and phosphorylation of AMPK, Akt and eNOS were significantly elevated in our exercise training groups. The results indicated that κ-OR-mediated signaling was involved in the cardioprotection induced by exercise training.

Exercise training as a type of intervention and its cardioprotective effects on the I/R myocardium constitute an important direction of research in exercise and the prevention and treatment of diseases. Exercise training improves the myocardial structure and cardiac pump function. However, the mechanism of cardioprotection from exercise training is not entirely clear. Previous research has shown that exercise training alleviated myocardial I/R injury and promoted cardiovascular function recovery [2,4,6,7,16,39]. In this study, eight weeks of aerobic training elicited a significant improvement in hemodynamic index and reductions of myocardial infarct size and myocardial cTnT. These results suggested that long-term aerobic training exerted broad effects of cardioprotection. Moreover, it is very interesting that acute exercise training was also observed to have similar effects. It is our preliminary conception that HE would exhibit stronger cardioprotection than ME, which is why we designed the three different exercise groups. However, the results were not in agreement with our anticipation. We believe that at least two factors are responsible for the lack of difference: one is statistical, that is, that more samples are needed for the study to produce a positive, significant difference; the other is that it is conceivable that we examined cardioprotection immediately after exercise, not after 24h or later, so the effects of these three groups could be similar. The different effects in the three exercise groups might have been appeared at some time, such as after 24h or later, of course, but this hypothesis requires further study.

κ-OR is the main subtype of opioid receptor in myocardial tissue, and it plays a role in regulating the cardiovascular system and protecting myocardial tissue against I/R injury [22,24,26,27]. In this study, we demonstrated that the expression of κ-OR was significantly elevated in the exercise training groups. In addition, exercise training alleviated cardiac dysfunction and decreased myocardial infarct size and serum cTnT release caused by I/R, and these effects of exercise training were blocked by nor-BNI. This observation provided a link between the known cardioprotective effect of exercise training and the κ-OR system. Moreover, it has been reported that other opioid receptor subtypes had similar effects to κ-OR. Miller et al. demonstrated that delta-OR partly caused cardioprotection against necrosis [30]. However, some studies have suggested that delta-OR but not κ-OR played a key role contributing to the cardioprotection conferred by endurance exercise. Borges et al indicated that delta-OR expression was increased after 4 days of an exercise training protocol [29]. In the present study, we examined κ-OR expression immediately after the training but not 24 hours later, and the mRNA level of the opioid receptor was increased immediately, in agreement with the previous study by Dickson et al [16]. As a result, in addition to delta-OR, the cardioprotection of exercise training also seems related to κ-OR stimulation via the κ-OR-mediated signaling pathway.

AMPK elicits important cardioprotective effects against I/R injury [40]. It has been clarified that activation of AMPK elevates energy support in tissue to alleviate the tissue injury caused by deficiency in energy [41]. Furthermore, exercise training increases the phosphorylation of AMPK to regulate the metabolism of myocardial tissue [35,36]. In this study, in agreement with previous studies [42,43,44], all three exercise training protocols with different intensities elevated the phosphorylation of AMPK. In ischemic hearts, activation of AMPK is an adaptive response, and it stimulates ATP production, which protects myocardial tissue from anoxia [32,33,34]. In this study, Compound C abolished the cardioprotective effect of exercise training on I/R myocardial tissue, which indicated that exercise training protected the myocardium via an AMPK-dependent pathway. We also observed the effects of Akt and eNOS in cardioprotection by exercise training on I/R myocardial tissue. Akt and eNOS, as the signaling transduction molecules, play important roles in I/R myocardial tissue. It was manifested in this study that exercise training increased the phosphorylation of Akt and eNOS, and activation of Akt stimulated eNOS and then increased NO production. This result was similar to previous studies [45,46,47,48]. The subsequent results showed that exercise training-induced cardioprotection improved cardiac function, and decreased myocardial infarct size and serum cTnT were reversed by Akt inhibitor and L-NAME. Thus, Akt and eNOS participated in the cardioprotection induced by exercise training. These data demonstrated that exercise training exerted cardioprotective effects via the Akt-eNOS signaling pathway. Generally, the results suggested that stimulation of κ-OR by exercise training exerted cardioprotective effects via the AMPK-Akt-eNOS signaling pathway.

The mechanism of opioid receptor-mediated cardioprotection is indeed more complicated. It is well known that there is an interaction between κ-OR and adrenergic receptors in the heart; further studies are needed to elucidate these complicated mechanisms.

## Study limitations

Recently, Arida et al reported that higher opioid receptor binding was observed in the acute exercise animal group, with the opposite findings in the chronic exercise group, indicating that there must have been a different influence between the acute or long-term exercise groups[49]. The present study investigated the changes in κ-OR expression and mediators immediately after the three types of exercise training; why exercise leads to similar changes in κ-OR expression and related signaling mediators remains unknown. The acclimation period in the AE group and a study repeated with 24h of cessation between vigorous exercise and experimentation are required.

## Conclusion

In summary, our present study demonstrated that exercise training protected cardiac function and reduced myocardial infarct size and serum cTnT in myocardial I/R, and these cardioprotective effects were mediated by the κ-OR-AMPK-Akt-eNOS signaling pathway.

## Supporting information

S1 Fig Representative photographs of triphenyltetrazolium chloride (TTC)-stained left ventricular tissue. (A) Sham; (B) I/R, 30 min ischemia and 120 min reperfusion; (C) I/R + ME, moderate intensity exercise for 8 wk before I/R; (D) I/R + HE, high intensity exercise for 8 wk before I/R; (E) I/R + AE, acute exercise before I/R; (F) I/R + ME+ nor-BNI; (G) I/R + HE + nor-BNI; (H) I/R + AE + nor-BNI; (I) I/R + ME + Compound C; (J) I/R + HE + Compound C; (K) I/R + AE + Compound C; (L) I/R + ME + Akt inhibitor; (M) I/R + HE + Akt inhibitor; (N) I/R + AE + Akt inhibitor; (O) I/R + ME + L-NAME; (P) I/R + HE + L-NAME; (Q) I/R + AE + L-NAME. In each study, nor-BNI (a selective κ-OR antagonist, 2.0 mg/kg), Compound C (an AMPK inhibitor, 20 mg/kg), Akt inhibitor (0.3 mg/kg) and L-NAME (an eNOS inhibitor, 30 mg/kg) were administered 20min before reperfusion. Left ventricular tissue was then processed and stained with TTC to determine viable (red) and nonviable (white) myocardium.
(TIF)

S2 Fig **Effects of exercise training with different intensity on expression of** κ**-OR (A), AMPK (B), Akt (C) and eNOS (D)**. All results are expressed as means ± SEM. ME, moderate intensity exercise for 8 wk; HE, high intensity exercise for 8 wk; AE, acute exercise. n = 8, **P<0.01 vs Control.
(TIF)

S1 File The original data of changes of cardiac hemodynamics during myocardial I/R. All results are expressed as means ± SEM. I/R, 30 min ischemia and 120 min reperfusion; I/R + ME, moderate intensity exercise for 8 wk before I/R; I/R + HE, high intensity exercise for 8 wk before I/R; I/R + AE, acute exercise before I/R; I/R + ME+ nor-BNI, moderate intensity exercise for 8 wk before I/R plus nor-BNI (2.0 mg/kg); I/R + HE + nor-BNI, high intensity exercise for 8 wk before I/R plus nor-BNI (2.0 mg/kg); I/R + AE + nor-BNI; acute exercise before I/R plus nor-BNI (2.0 mg/kg); I/R + ME + Compound C, moderate intensity exercise for 8 wk before I/R plus Compound C (20 mg/kg); I/R + HE + Compound C, high intensity exercise for 8 wk before I/R plus Compound C (20 mg/kg); I/R + AE + Compound C, acute exercise before I/R plus Compound C (20 mg/kg); I/R + ME + Akt inhibitor, moderate intensity exercise for 8 wk before I/R plus Akt inhibitor (0.3 mg/kg); I/R + HE + Akt inhibitor, high intensity exercise for 8 wk before I/R plus Akt inhibitor (0.3 mg/kg); I/R + AE + Akt inhibitor, acute exercise before I/R plus Akt inhibitor (0.3 mg/kg); I/R + ME + L-NAME, moderate intensity exercise for 8 wk before I/R plus L-NAME (30 mg/kg); I/R + HE + L-NAME, high intensity exercise for 8 wk before I/R plus L-NAME (30 mg/kg); I/R + AE + L-NAME, acute exercise before I/R plus L-NAME (30 mg/kg). n = 8.
(SAV)

S2 File The original data of effects of exercise training on cTnT in serum after I/R. All results are expressed as means ± SEM. I/R, 30 min ischemia and 120 min reperfusion; I/R + ME, moderate intensity exercise for 8 wk before I/R; I/R + HE, high intensity exercise for 8 wk before I/R; I/R + AE, acute exercise before I/R; I/R + ME+ nor-BNI, moderate intensity exercise for 8 wk before I/R plus nor-BNI (2.0 mg/kg); I/R + HE + nor-BNI, high intensity exercise for 8 wk before I/R plus nor-BNI (2.0 mg/kg); I/R + AE + nor-BNI; acute exercise before I/R plus nor-BNI (2.0 mg/kg); I/R + ME + Compound C, moderate intensity exercise for 8 wk before I/R plus Compound C (20 mg/kg); I/R + HE + Compound C, high intensity exercise for 8 wk before I/R plus Compound C (20 mg/kg); I/R + AE + Compound C, acute exercise before I/R plus Compound C (20 mg/kg); I/R + ME + Akt inhibitor, moderate intensity exercise for 8 wk before I/R plus Akt inhibitor (0.3 mg/kg); I/R + HE + Akt inhibitor, high intensity exercise for 8 wk before I/R plus Akt inhibitor (0.3 mg/kg); I/R + AE + Akt inhibitor, acute exercise before I/R plus Akt inhibitor (0.3 mg/kg); I/R + ME + L-NAME, moderate intensity exercise for 8 wk before I/R plus L-NAME (30 mg/kg); I/R + HE + L-NAME, high intensity exercise for 8 wk before I/R plus L-NAME (30 mg/kg); I/R + AE + L-NAME, acute exercise before I/R plus L-NAME (30 mg/kg). n = 8,
(SAV)
