# Supplementary figures and images for: κ-opioid receptor is involved in the cardioprotection induced by exercise training
Source: PLoS One. 2017 Mar 16;12(3):e0170463. doi: 10.1371/journal.pone.0170463 (PMC5354247; doi:10.1371/journal.pone.0170463)

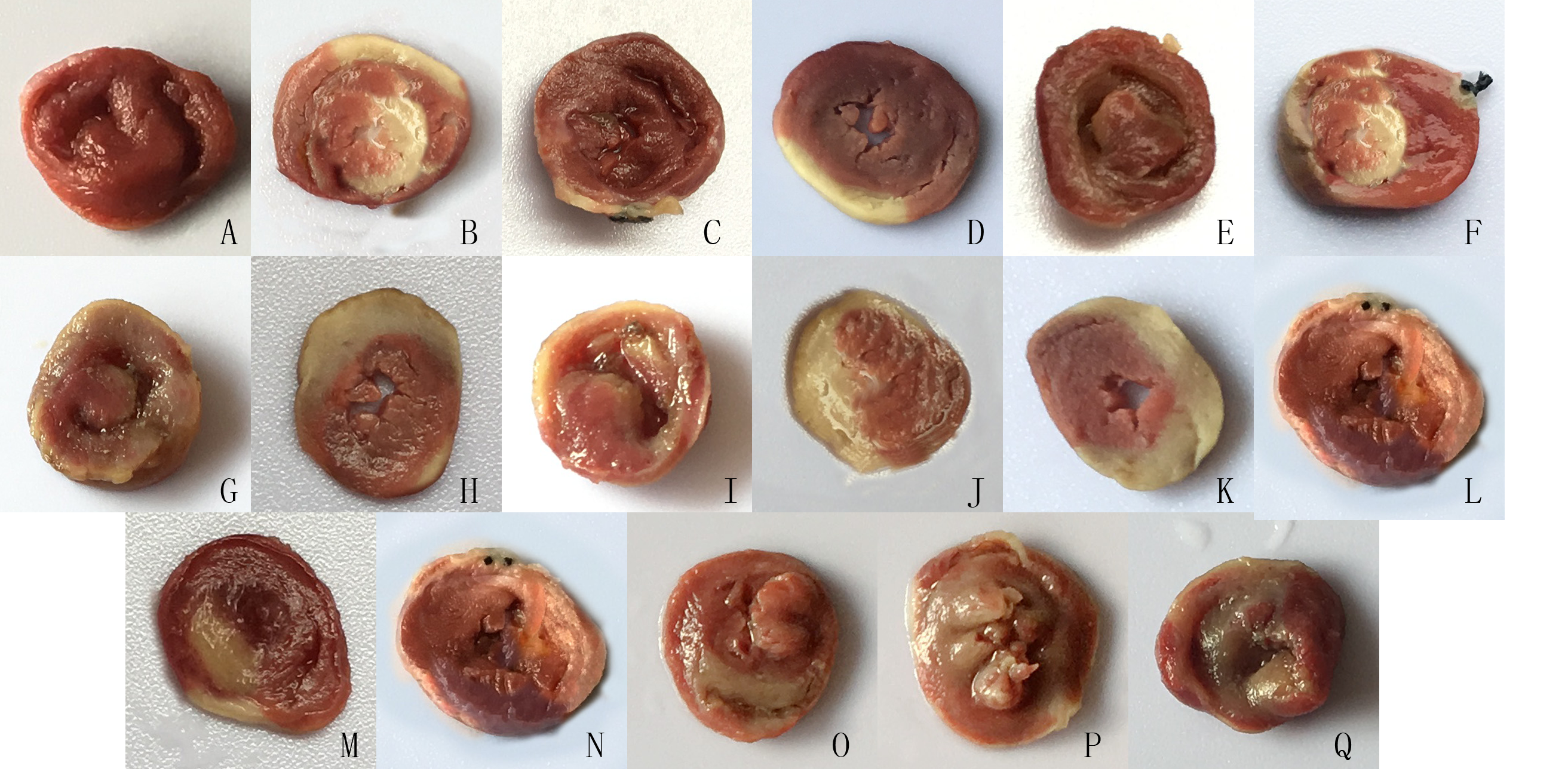

Supplement: S1 Fig — (A) Sham; (B) I/R, 30 min ischemia and 120 min reperfusion; (C) I/R + ME, moderate intensity exercise for 8 wk before I/R; (D) I/R + HE, high intensity exercise for 8 wk before I/R; (E) I/R + AE, acute exercise before I/R; (F) I/R + ME+ nor-BNI; (G) I/R + HE + nor-BNI; (H) I/R + AE + nor-BNI; (I) I/R + ME + Compound C; (J) I/R + HE + Compound C; (K) I/R + AE + Compound C; (L) I/R + ME + Akt inhibitor; (M) I/R + HE + Akt inhibitor; (N) I/R + AE + Akt inhibitor; (O) I/R + ME + L-NAME; (P) I/R + HE + L-NAME; (Q) I/R + AE + L-NAME. In each study, nor-BNI (a selective κ-OR antagonist, 2.0 mg/kg), Compound C (an AMPK inhibitor, 20 mg/kg), Akt inhibitor (0.3 mg/kg) and L-NAME (an eNOS inhibitor, 30 mg/kg) were administered 20min before reperfusion. Left ventricular tissue was then processed and stained with TTC to determine viable (red) and nonviable (white) myocardium. (TIF) [file pone.0170463.s001.tif]

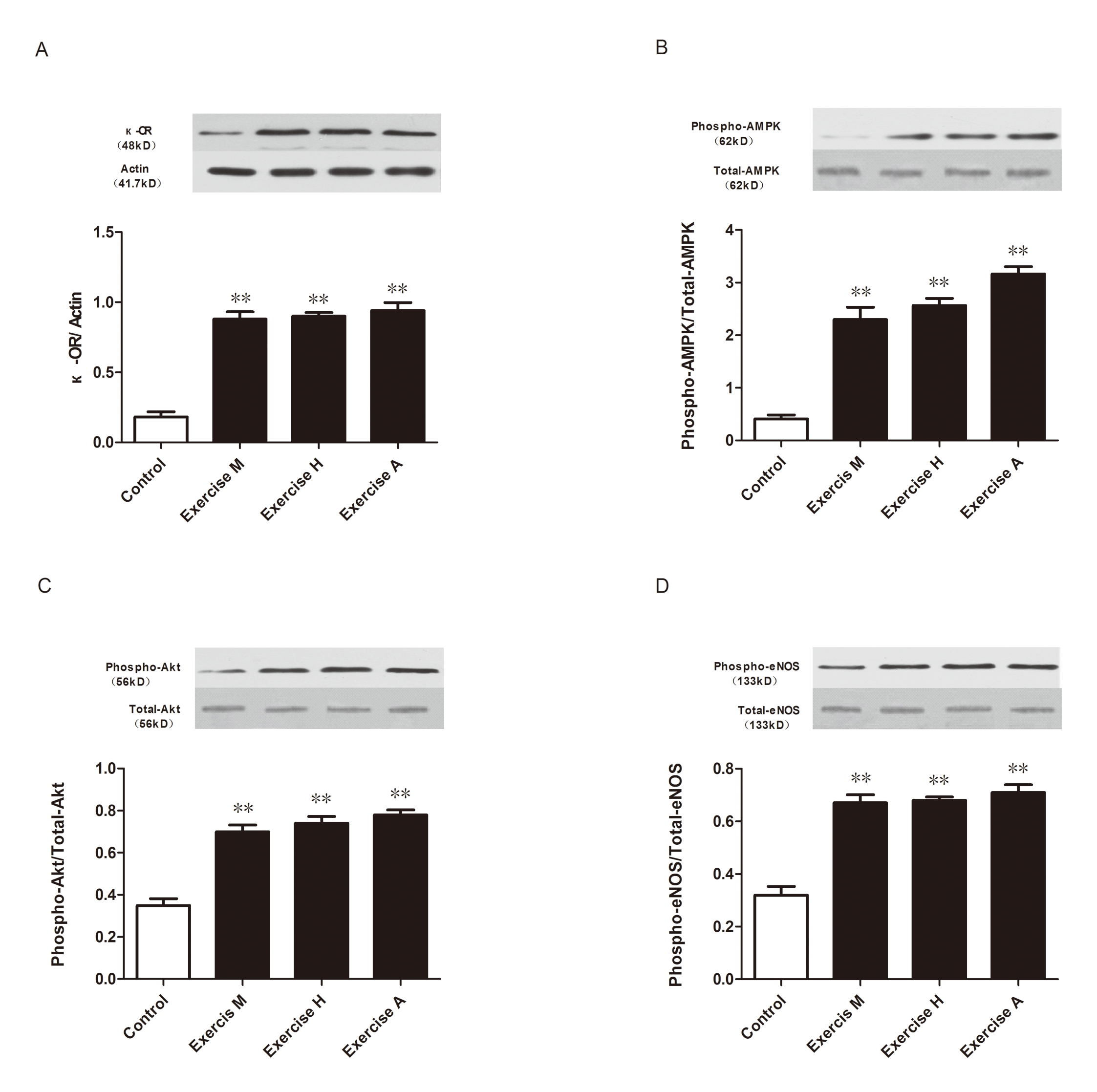

Supplement: S2 Fig — Effects of exercise training with different intensity on expression of κ-OR (A), AMPK (B), Akt (C) and eNOS (D). All results are expressed as means ± SEM. ME, moderate intensity exercise for 8 wk; HE, high intensity exercise for 8 wk; AE, acute exercise. n = 8, **P<0.01 vs Control. (TIF) [file pone.0170463.s002.tif]
